# AI‐Assisted Detection of Early Gastric Cancer via Visualization of Mucosal Acidity Compromise During Endoscopy

**DOI:** 10.1002/advs.202504932

**Published:** 2025-10-20

**Authors:** Huihui Yan, Zongkuo Li, Jing Zhao, Lei Su, Ziyi Jin, Weiyi Zhao, Rong Duan, Suhongrui Zhou, Lingling Wang, Jianshan Mao, Xinliang Lu, Weihao Gai, Yang Du, Qin Du, Cheng Fang, Yiming Zhao, Yan You, Jianting Cai, Cong Li

**Affiliations:** ^1^ Department of Gastroenterology The Second Affiliated Hospital Zhejiang University School of Medicine Hangzhou 310009 China; ^2^ School of Pharmaceutical Sciences Fudan University MOE Key Laboratory of Smart Drug Delivery MOE Innovative Center for New Drug Development of Immune Inflammatory Diseases State Key Laboratory of Medical Neurobiology Shanghai 201203 China; ^3^ CAS Key Laboratory of Molecular Imaging Institute of Automation Chinese Academy of Sciences Beijing 100190 China; ^4^ Department of Pathology The Second Affiliated Hospital Zhejiang University School of Medicine Hangzhou 310009 China; ^5^ Department of Child Healthcare Jiangbei District Maternal and Child Health and Family Planning Service Center Ningbo 315020 China; ^6^ Department of Pharmacology School of Pharmacy Fudan University Shanghai 201203 China

**Keywords:** artificial intelligence, early gastric cancer, endoscopy, gastric acidity, surface‐enhanced Raman scattering

## Abstract

Accurate localization of early gastric cancer (EGC) remains challenging due to its morphological resemblance to gastritis. This study presents an artificial intelligence (AI)‐assisted bedside diagnostic system to enhance EGC detection by visualizing gastric mucosal acidity. The ATPase H^+^/K^+^ transport β subunit (ATP4B), a key regulator of acid secretion, is progressively downregulated in gastric mucosal atrophy and intestinal metaplasia, and significantly reduced in EGC. A surface‐enhanced Raman scattering (SERS) microarray is developed to map mucosal pH in 50 patient specimens (1,516 points), with founding compared to pathological images. A multi‐model neural network is trained and validated internally on data from 40 patients (1,127 points) and externally validated on 10 patients (389 points). Using an optimal pH threshold of 6.845, the system achieved a strong correlation (R^2^ = 0.79) and low error (SSE = 71.83). External validation demonstrated 87.79% sensitivity, 85.04% specificity, 86.89% accuracy, and a κ score of 0.71. This system detected mild pH shifts in atrophic gastritis with intestinal metaplasia, but marked increases with EGC onset, and is able to predict inflammation prior to pathology confirmation. By integrating pH mapping with morphological features, this approach enables precise EGC localization, improves guidance for endoscopic submucosal dissection (ESD), and reduces false‐positive diagnoses.

## Graphical abstract

An AI‐assisted SERS system is developed to rapidly and reliably detect early gastric cancer by visualizing gastric mucosal acidity, enhancing endoscopic differentiation, improving guidance for submucosal dissection, and reducing false‐positive diagnoses.

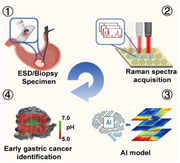

## 1 Introduction

Gastric cancer poses a major global health challenge, ranking fifth in both incidence and cancer‐related mortality worldwide, with the absolute number of new cases projected to continue rising.^[^ ^1^, ^2^ ^]^ Early gastric cancer (EGC), confined to the mucosa or submucosa, has a favorable prognosis, with a 5‐year survival rate exceeding 90%.^[^ ^3^ ^]^ In stark contrast, the survival rate drops to just 20‒30% once the disease progresses to an advanced stage.^[^ ^4^ ^]^ These statistics underscore the critical importance of early detection in improving patient outcomes.

Endoscopic submucosal dissection (ESD) is the preferred treatment for EGC, offering en bloc resection with minimal reliance on piecemeal removal or surgical excision. This technique offers several advantages, including minimal invasiveness, quick recovery, and preservation.^[^ ^5^ ^]^ However, precise localization of cancer tissue during ESD is crucial to avoid overtreatments, incomplete resections, and local recurrences. Inaccurate targeting may also lead to complications such as delayed bleeding (7‒15.6%) and elevated perforation rates (3.6‒4.5%) associated with unnecessary tissue resection.^[^ ^6^, ^7^, ^8^ ^]^ Therefore, both early detection and accurate lesion identification are essential for achieving successful clinical outcomes.

Identifying EGC from gastritis remains a significant challenge due to their morphological similarities under endoscopy. This difficulty is especially pronounced in cases of *Helicobacter pylori*‐induced gastritis, which often presents with irregular mucosal elevation and erythema.^[^ ^9^, ^10^ ^]^ As a result, the diagnostic accuracy for EGC is relatively low, averaging ≈60%.^[^ ^9^, ^11^ ^]^ Even after *H. pylori* eradication, EGC may still mimic gastritis, further complicating diagnosis.^[^ ^10^ ^]^ While advancements in endoscopic technology, such as high‐definition white‐light endoscopes (HD‐WLE), have improved lesion detection through enhanced spatial resolution, 20‒25% of EGC cases remain undetected.^[^ ^12^ ^]^ Additional technologies, including virtual chromoendoscopy, narrow‐band imaging (NBI), and blue laser imaging, have improved visualization of tumor‐associated vascular irregularities and aid in EGC localization.^[^ ^13^ ^]^ However, even with a magnifying endoscope combined with NBI (ME‐NBI), diagnostic accuracy remains suboptimal, with over 12% of EGC cases still misdiagnosed.^[^ ^14^ ^]^ Moreover, these techniques require substantial operator expertise, and inter‐observer variability remains high. These limitations highlight the urgent need for new diagnostic strategies that go beyond morphology‐based assessments and instead target molecular alternations to improve the identification and localization of EGC.

**Figure 1:**
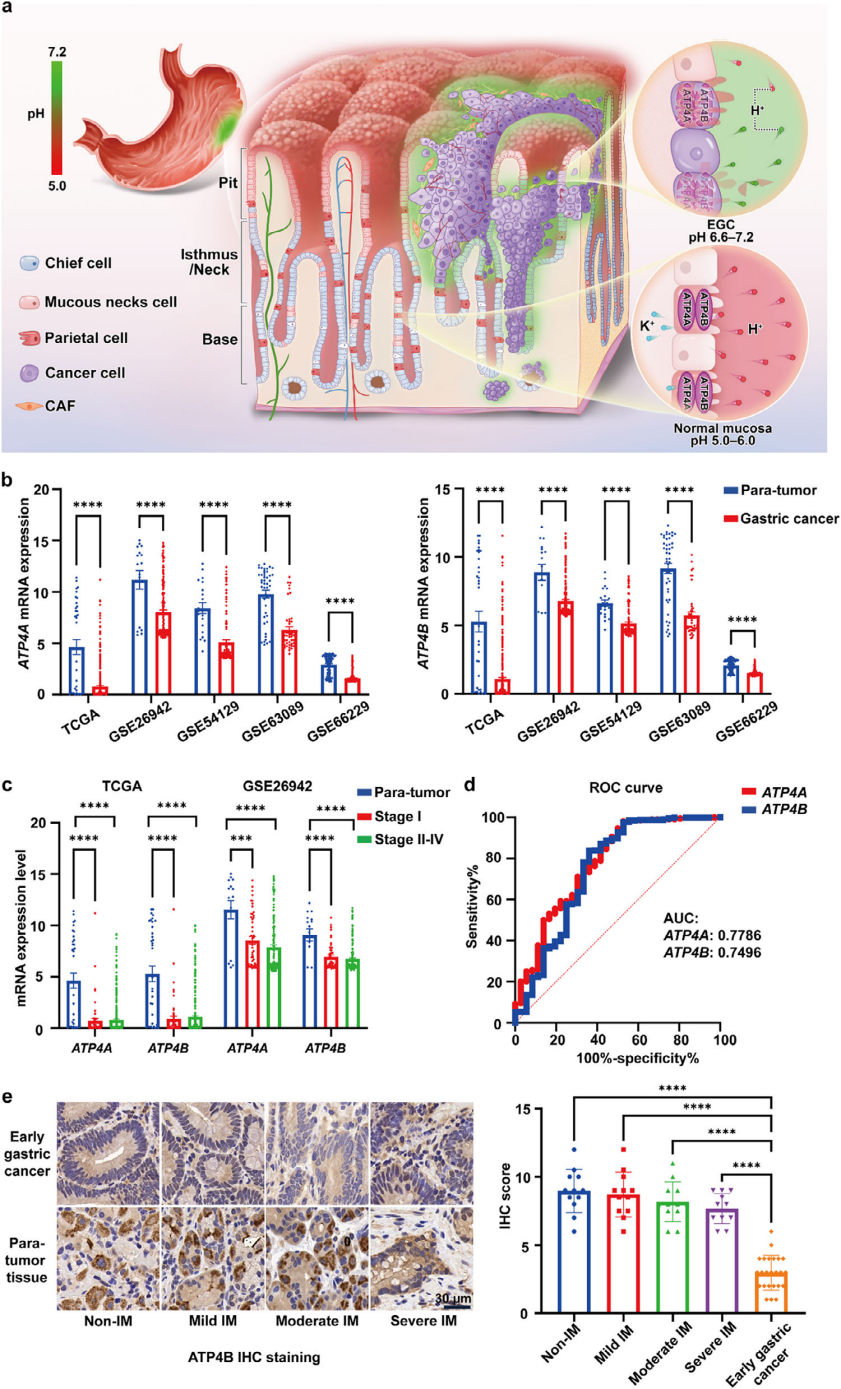
Compromised gastric mucosal acidity as a biomarker for EGC. a) Schematic illustration: In normal gastric mucosa, parietal cells within the glandular ducts secrete protons through ATP4A/B channels, maintaining an acidic environment (pH 5.0–6.0). In regions affected by EGC, damage to parietal cells results in compromised acidity (pH 6.6–7.2). b) mRNA expression of *ATP4A* and *ATP4B* in tumor and para‐tumor tissue samples across multiple datasets (TCGA, GSE26942, GSE54129, GSE63089, and GSE66229). c) Comparison of *ATP4A* and *ATP4B* mRNA expression in early‐stage (stage I) and late‐stage (stage II‐IV) gastric cancer using data from TCGA and GSE26942. d) Analysis of decreased *ATP4A* and *ATP4B* expression in gastric tumor specimens from the TCGA database, highlighting their high sensitivity and specificity in gastric cancer diagnosis. e) Immunohistochemical staining of ATP4B protein in human gastric tissue specimens with different pathological classifications. Scale bar: 30 µm. Data are presented as mean ± S.D. Statistical significance was determined using an unpaired *t*‐test and one‐way analysis of variance (ANOVA), with a *p*‐value of < 0.05 considered significant.

Recent studies have reported a significant decrease in the expression of ATPase H^+^/K^+^ transport β subunit (ATP4B) in gastric cancer,^[^ ^15^, ^16^, ^17^, ^18^, ^19^ ^]^ acting as a potential biomarker for malignant transformation in the gastric mucosa.^[^ ^19^ ^]^ Animal models have further demonstrated that mice with reduced *ATP4B* mRNA expression exhibit correspondingly lower gastric acid levels.^[^ ^20^ ^]^ These findings imply that the compromised gastric acidity due to *ATP4B* downregulation may serve as a functional indicator of gastric cancer, including EGC (**Figure** **1**a).

Raman scattering is a light‐matter interaction in which incident photons undergo energy shifts after interacting with molecular vibrations, providing detailed molecular fingerprints with minimal sample preparation and reduced water interference.^[^ ^21^ ^]^ This makes Raman spectroscopy a powerful technique for the identification and characterization of biomolecules.^[^ ^22^ ^]^ Building on this principle, surface‐enhanced Raman scattering (SERS) retains the molecular specificity of conventional Raman spectroscopy while significantly enhancing signal intensity through plasmonic excitation on metallic nanostructures.^[^ ^23^, ^24^ ^]^ This signal amplification enables SERS to detect and differentiate tumors with high sensitivity, specificity, and reliability, owing to molecular variations.^[^ ^25^ ^]^

In this work, we confirmed ATP4B as a molecular marker for EGC and proposed that acidity compromise in the gastric mucosa serves as a spatial indicator for EGC location. To visualize these pH alternations, we developed a SERS microarray platform, which was first validated in animal models. Furthermore, we integrate this platform with an artificial intelligence (AI)‐assisted, multi‐model neural network capable of real‐time data processing during endoscopy. This system enables the precise differentiation of EGC from gastritis by detecting subtle pH changes in the gastric mucosa, thereby improving diagnostic accuracy and localization during endoscopic procedures.

## 2 Results

### 2.1 ATP4B Downregulation Associated Gastric Acidity Compromise Serves as a Potential Biomarker for EGC

Recent studies have found that the loss of ATP4 (H^+^/K^+^ ATPase), a marker of parietal cells, is a characteristic of gastric cancer.^[^ ^15^, ^16^, ^17^, ^18^, ^19^ ^]^ Exploring its potential as a biomarker, we analyzed *ATP4* mRNA expression in gastric cancer patients using data from the Cancer Genome Atlas (TCGA) and Gene Expression Omnibus (GEO). We observed a significant down‐regulation of *ATP4A* (ATPase H^+^/K^+^ transporting alpha subunit) and *ATP4B* mRNA in cancerous tissues compared to non‐cancerous tissues (Figure 1b). This downregulation was evident in the early stages of gastric cancer and did not decrease significantly with disease progression (^****^ *p* < 0.0001, ^***^ *p* < 0.001, Figure 1c). The areas under the curve (AUCs) were 0.7786 and 0.7496 for *ATP4A* and *ATP4B* mRNA, respectively (95% CI, 0.6885‒0.8687 and 0.6452‒0.8540, respectively) (Figure 1d), suggesting its potential utility as a biomarker for gastric cancer. To further verify the level of ATP4B in gastric cancer, we analyzed 24 non‐tumor gastric mucosal biopsy specimens and the tumor‐adjacent tissues of 24 ESD specimens, with four different stages of gastritis with intestinal metaplasia (IM), 6 specimens for each stage. ATP4B level decreases progressively with the advancement of gastric mucosal atrophy and intestinal metaplasia, and is markedly down‐regulated during the process of carcinogenesis (Figure 1e; Figure S1, Supporting Information), providing further evidence supporting ATP4B as a sensitive and reliable diagnostic marker for gastric cancer.

Given that ATP4B plays a crucial role in the synthesis and secretion of H^+^ in parietal cells, which is essential for maintaining the acidic environment in the stomach, the reduced expression of ATP4B may indicate a compromised acidity in gastric cancer tissues compared to adjacent non‐cancerous tissues.

### 2.2 A SERS Microarray Platform for pH Mapping in Biopsy and ESD Specimens

**Figure 2:**
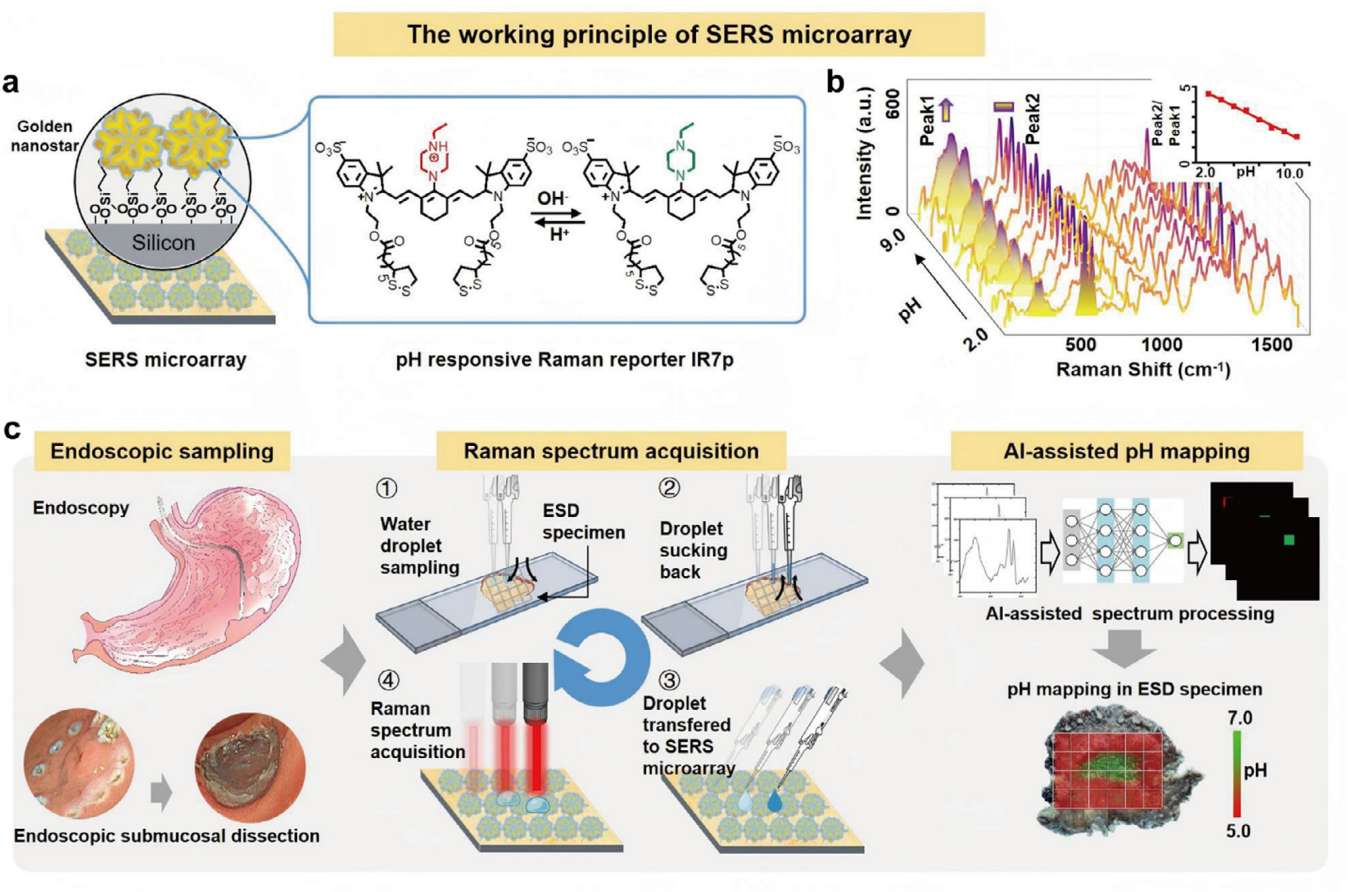
Establishment of a SERS microarray for spatial pH profiling in gastric mucosal specimens. a) Preparation of a pH ratio‐metric SERS microarray. The surface of a silicon wafer is functionalized with primary amines, followed by conjugation with nano‐stars (average diameter 70 nm). These nano‐stars are further functionalized with the pH‐responsive Raman reporter IR7p, enabling pH‐sensitive Raman spectra. b) pH‐dependent Raman spectra from the SERS microarray. While Peak1 intensity increases with pH, the intensity of Peak2 keeps unchanged as an internal reference. c) Localization of tumor margins of EGC via pH mapping. Approximately 0.5 µL of pure water is aspirated and applied to the endoscopic specimen for 2.0 s. After retraction, the water droplet is then added to the SERS microarray. Raman spectra are collected using a Raman scanner with a 785 nm laser. This process is repeated at multiple points to generate a comprehensive set of Raman spectra, which are then analyzed using a spectral processing algorithm to create spatial pH profiling of the biopsy and ESD specimens.

To explore whether the absence of ATP4B in gastric cancer alters pH levels within the tumor and whether these pH shifts serve as a reliable marker for identifying cancerous tissue, it is essential to first establish a method to precisely measure gastric mucosa pH. To achieve this, a SERS microarray‐based strategy was developed (**Figure** **2**a), with a detailed description of the preparation process of the SERS microarray chips provided in Figure S2 (Supporting Information). Scanning electron microscopy (SEM) analysis reveals a uniform distribution of gold nanospheres and nano‐stars on the chip surface, with the nanoparticles exhibiting a consistent diameter of 67.95 ± 5.99 nm, corresponding to a relative standard deviation (RSD) of less than 9%, and an even spatial distribution of 101.2 ± 5.391/µm^2^ (Figure S3, Supporting Information). This high uniformity ensures consistent local electromagnetic enhancement across the sensing area. Furthermore, the SERS microarray chips show excellent reproducibility across different batches in response to solutions of varying pH values (Figure S4, Supporting Information). The Raman reporter **IR7p** demonstrates pH‐dependent absorption. Utilizing a handheld Raman scanner with a 785 nm laser, we collected pH‐dependent Raman spectra from the microarray following the introduction of saline droplets at various pH levels. Notably, while Peak 1 at 303 cm^−1^ intensifies with increasing pH, Peak 2 at 520 cm^−1^ remains stable (Figure 2b). A conventional custom spectral processing algorithm automatically calculates the ratio of Raman Peak 2 to Peak 1, which ranges from 1.7 to 4.5 as the pH decreases from 9.0 to 2.0. This relationship follows a linear equation: y = −0.4201x + 5.3972 (R^2^ = 0.9904). The procedure for utilizing the SERS microarray to map pH in biopsy/ESD samples is shown in Figure 2c.

### 2.3 SERS Microarray Enables Precise Identification of Orthotopic EGC Xenografts in Mice

**Figure 3:**
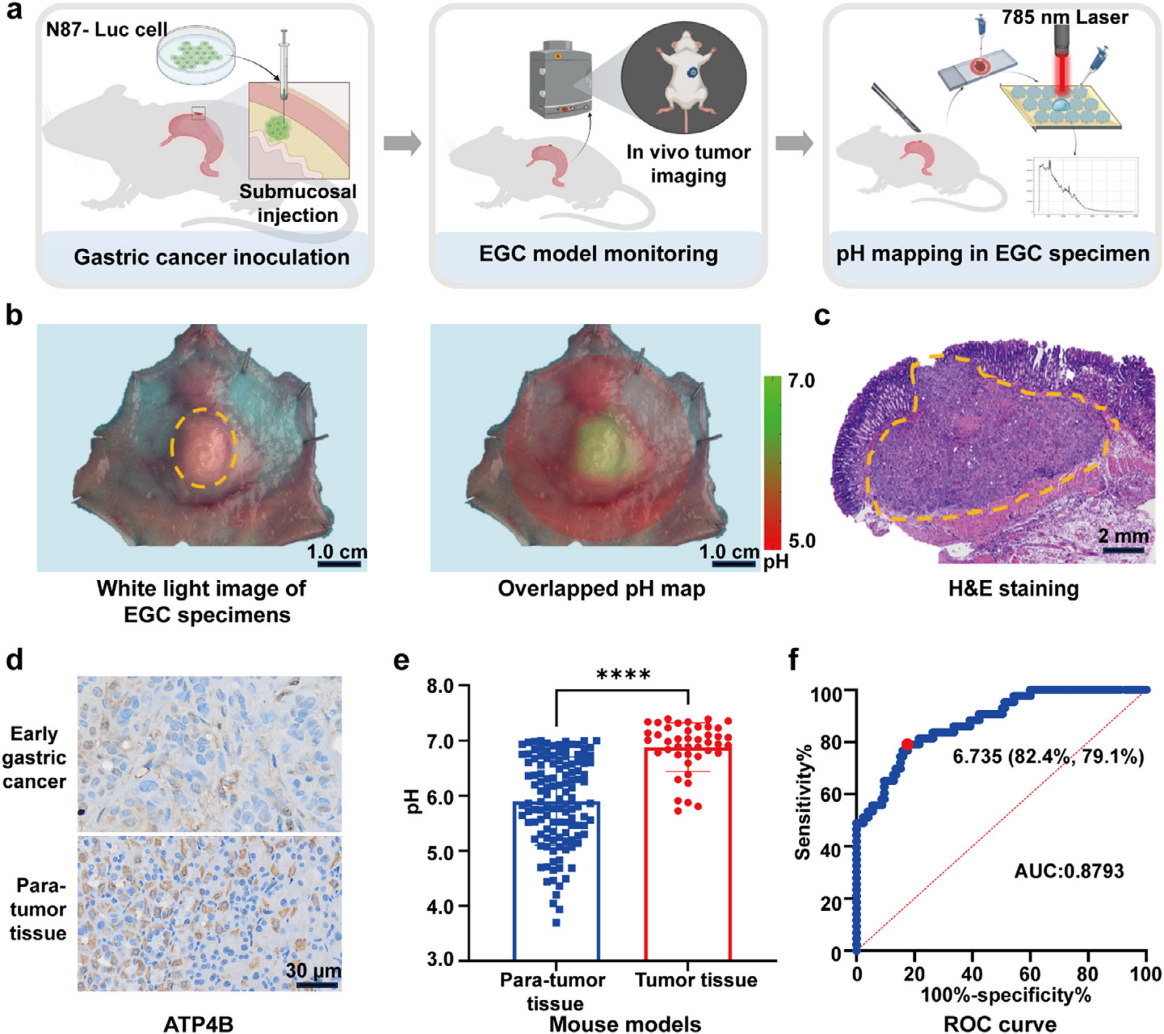
SERS microarray system identifies gastric tumor xenograft in mouse mucosal specimens. a) Establishment of an orthotopic EGC xenograft in mouse models. Human gastric N87‐Luc cancer cells are injected into the submucosa of a nude mouse. The pH map of the resected specimen is obtained using the SERS microarray system. b) White light image (left panel) showing EGC (indicated by a yellow dotted circle) and an overlaid pH map (right) of the excised EGC specimen. Scale bar: 1.0 cm. c) H&E staining of the EGC tumor xenograft, with the tumor region outlined by a yellow dotted line. d) Immunohistochemical staining of ATP4B in tumor and para‐tumor gastric tissues. Scale bar: 30 µm. e) pH measurements of the tumor and surrounding gastric tissue in excised specimens (*N* = 10, total points = 168; tumor vs para‐tumor: 43 vs 125, *p* < 0.0001). f) ROC curve analysis for determining the optimal pH threshold to distinguish tumor tissue. The optimal threshold is 6.735, with a sensitivity of 82.4% (95% CI, 0.7479‒0.8808), specificity of 79.3% (95% CI, 0.6479‒0.8858), and an AUC of 0.8793 (95% CI, 0.8211‒0.9374). Data are presented as mean ± S.D. Statistical significance was determined using an unpaired *t*‐test, with a *p*‐value of < 0.05 considered significant.

To assess the feasibility of SERS microarray in locating EGC by monitoring gastric mucosa pH, we created an orthotopic EGC xenograft by injecting human NCI‐N87‐luc gastric cancer cells into nude mice (**Figure** **3**a). Tumor growth was monitored via optical imaging (Figure 3a; Figure S5, Supporting Information). After 14 days, the gastric tumor and adjacent mucosa were excised, and their pH maps, with a 3.0 mm × 3.0 mm resolution, were determined using SERS microarray (Figure 3a,b). H&E (hematoxylin and eosin) staining confirmed the localization of submucosal gastric cancer (Figure 3c). Immunohistochemical staining in the mouse model mirrored ATP4B protein level observed in human EGC (Figure 3d). pH assessments revealed significant differences in acidity between tumor and para‐tumor tissues (*p* < 0.0001, Figure 3e). Specifically, the maps showed weakly acidic areas (pH: 6.879 ± 0.4403) aligning with tumor locations, while adjacent normal tissues exhibited strongly acidic zones (pH: 5.903 ± 0.8000). Receiver operating characteristic (ROC) curve analysis yielded an AUC of 87.93% (95% CI, 0.8211‒0.9374), with a critical pH of 6.735 (Figure 3f). In addition, we also applied different gastric cancer cells (Human MKN‐45 and AGS) to establish orthotopic EGC xenografts. As shown in Figure S6a‒c (Supporting Information), in these tumor models, pH assessment revealed significant differences (*p* < 0.0001) between tumor and para‐tumor tissues. ROC curve analysis yielded AUCs of 86.58% (95% CI: 0.7989‒0.9327) and 85.92% (95% CI: 0.7898‒0.9286), with corresponding cut‐off pH values of 6.574 and 6.224, respectively (Figure S6d, Supporting Information). The animal models constructed by these three cell lines show similar results. These findings highlight the efficacy of the SERS microarray strategy in diagnosing EGC within surrounding non‐cancerous mucosa.

### 2.4 The SERS Microarray Enables Precise Localization of EGCs in Patients

**Figure 4:**
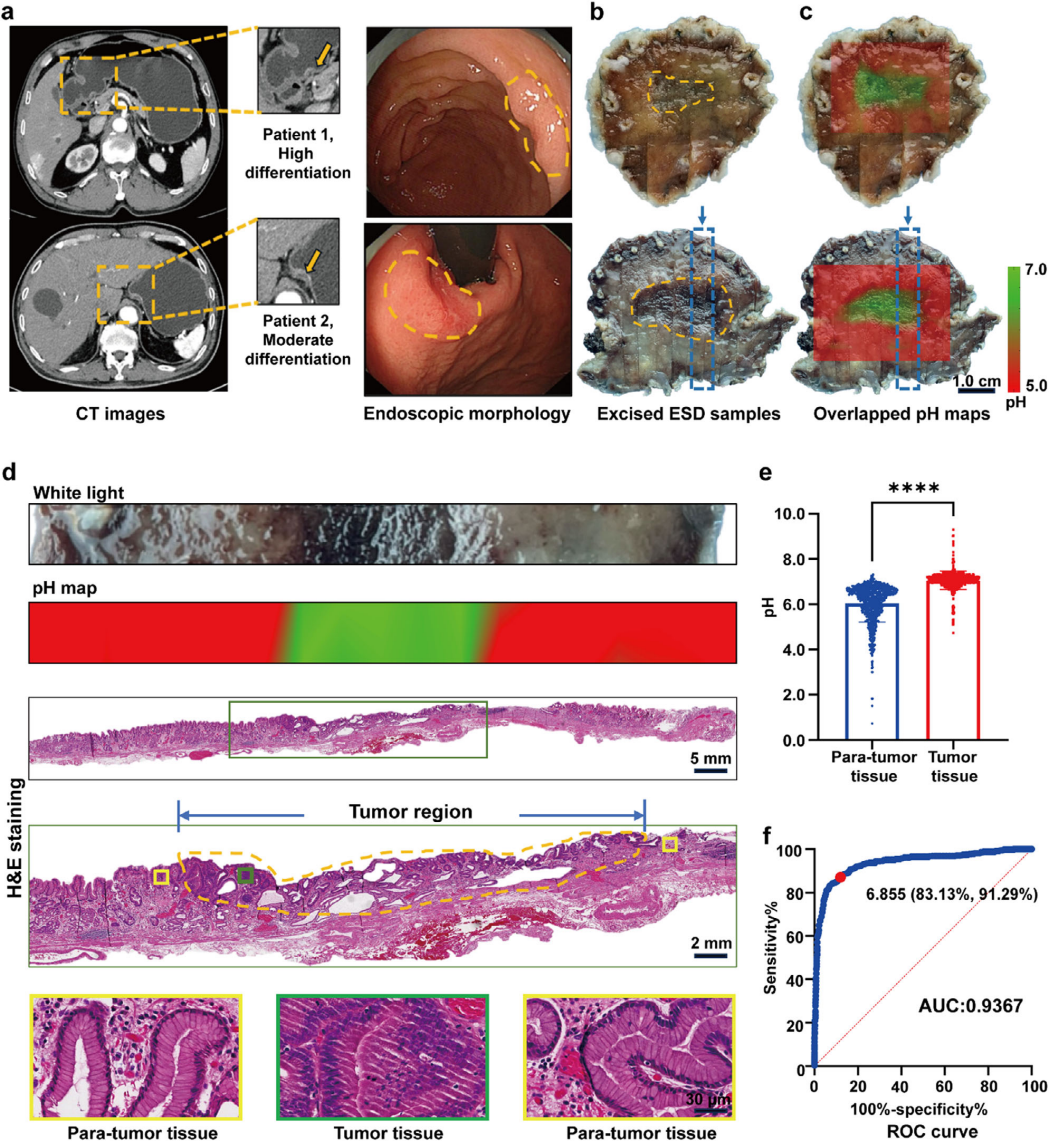
Spatial correlation between EGC and compromised acidity in patient samples. a) CT images of a patient showing the location of EGC (left panel). The middle panel provides an enlargement of the regions within the yellow dotted boxes, with yellow arrows highlighting suspected lesions. The right panel displays representative endoscopic images with yellow dotted lines outlining the EGC lesion boundaries. b) White light restoration maps of the ESD specimens with yellow dotted circles marking the EGC boundaries as confirmed by pathological examination. c) Overlap of the white light restoration maps and pH topographic maps generated by the SERS microarray. Green and red regions represent faintly acidic and strongly acidic areas, respectively. The upper panels of b & c correspond to a highly differentiated gastric cancer in ESD specimen, while the lower panels show a moderately differentiated gastric cancer. Scale bar: 1.0 cm. d) The upper row shows one strip of excised sample tissue from panel b (blue dotted box), while the second row displays the corresponding pH map. The third row verifies the tumor margins through H&E staining. The final two rows show enlarged images: the green box indicates the tumor, and the yellow box highlights the para‐tumor gastric mucosa. Scale bar: from top to bottom are 5.0 mm, 2.0 mm, and 30 µm. e) pH differences between tumor and para‐tumor gastric tissues in ESD samples from 50 patients (total points = 1516; tumor vs para‐tumor: 575 vs 941, *p* < 0.0001). The distribution of 1516 measurement points across 50 samples is well‐balanced. f) ROC curve analysis based on pH values from ESD specimens. The optimal threshold value is 6.855, with a corresponding AUC of 0.9367 (95% CI, 0.9229‒0.9504). Data are presented as mean ± S.D. Statistical significance was determined using an unpaired *t*‐test, with a *p*‐value of < 0.05 considered significant.

Animal experiment efficacy predicts promising potential for our endoscopic application. To reinforce the viability of the SERS microarray strategy we developed, 50 ESD samples with pathologically confirmed EGC were employed to assess acidity disparities between tumor and adjacent gastric mucosa. Computed tomography (CT) scans of EGC lesions showed minimal abnormalities, with original images on the left, magnified views of the lesions in the middle, and yellow arrows highlighting subtle changes. Endoscopic views, outlined by yellow dotted curves, showed minimal structural and color variations (**Figure** **4**a). To measure pH values on fresh gastric mucosal lesion specimens within a 5‐min timeframe, the point block method was employed (3.0 mm × 3.0 mm) (Figure 4b). Specifically, ≈36 measurement points were selected within a 2.0 × 2.0 cm^2^ area, from which a pH topographic graph was generated (Figure 4c). As seen, the tumor region in the area marked with a blue dotted box in Figure 4b and confirmed pathologically in Figure 4d, precisely matched the areas depicted on the pH map (Figure 4c). The upper panels of Figure 4b,c show a highly differentiated gastric cancer ESD specimen, while the lower panels show a moderately differentiated early gastric cancer ESD specimen. Our data showed that there was no significant difference in tumor region pH between highly differentiated and moderately differentiated EGC specimens (Figure S7, Supporting Information). In addition, a notable pH difference was observed between tumor and para‐tumor gastric mucosa in ESD samples from 50 patients (7.056 ± 0.4138 vs 6.046 ± 0.8352, *p* < 0.0001) (Figure 4e). ROC analysis using 941 pH measurements from para‐tumor and 575 from tumor areas across 50 samples yielded an AUC of 93.67% (95%CI, 0.9229‒0.9504), with the key pH value for differentiation being 6.855 (Figure 4f). These findings indicate that the SERS microarray can differentiate between tumor and non‐malignant sites in fresh gastric ESD samples within 5 min of Raman signal acquisition, and a pH map of a 2.0 × 2.0 cm^2^ specimen can be obtained after at least 30 min of standard curve measurements and conventional custom software algorithm calculations. Further analysis of the data reveals no notable difference in mucosal pH readings across different stages of atrophic gastritis with intestinal metaplasia. However, a marked elevation in pH levels becomes apparent upon the development of EGC (Figure S8, Supporting Information).

### 2.5 AI‐Assisted Prediction for Rapid and Accurate EGC Differentiation

**Figure 5:**
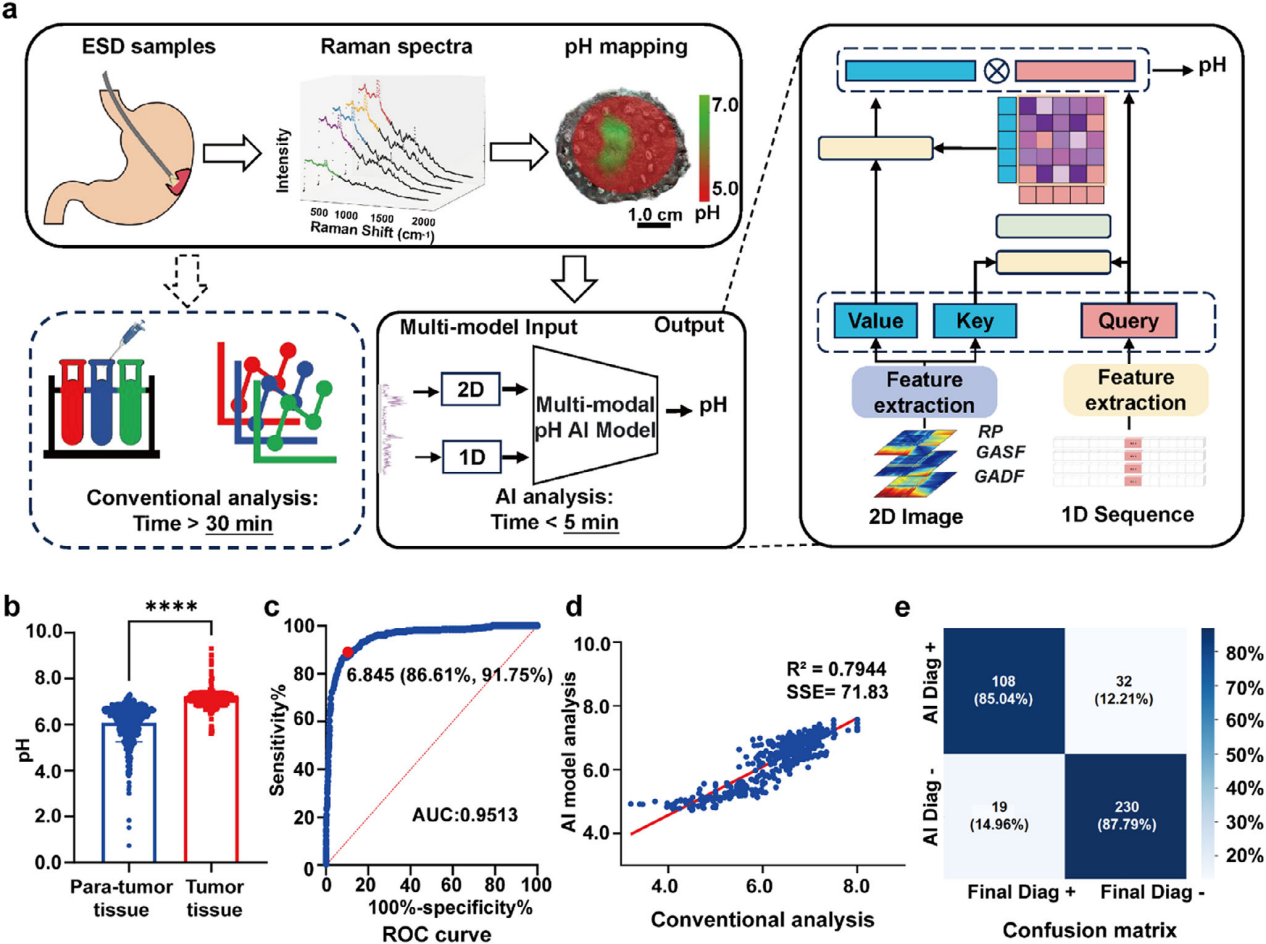
AI‐integrated SERS microarray for precise delineation of EGC. a) The multimodal pH prediction model extracts features from both 1D Raman sequences and 2D Raman images. The 2D Raman image is formed by concatenating three types of data‐Recurrence Plot (RP), Gramian Angular Summation Field (GASF), and Gramian Angular Difference Field (GADF)‐which are derived from the sequence self‐similarity, local similarity, and angular difference of the Raman spectrum. The Convolutional Neural Network and fully connected layers are used to capture 2D and 1D features, respectively. Then, the Co‐Attention mechanism is applied to interact with the information from both modalities. And the fused features are used to predict the pH value. b) Comparison of pH values in tumor regions versus adjacent gastric mucosa (*N* = 40, total points = 1127, *p* < 0.0001). c) ROC curve based on pH values from ESD specimens in the training and validation set of EGC patients. The optimal threshold value is 6.845, yielding an AUC of 95.13% (95%CI, 0.9386‒0.9639). d) Performance comparison between the deep learning model and a nonlinear regression model, with AI model achieving an R^2^ value of 0.7944 and an SSE of 71.83. e) Confusion matrix illustrating the diagnostic performance of AI‐assisted SERS microarray in differentiating benign and malignant gastric tissues (total points = 389). Sensitivity, 85.04%; Specificity, 87.79%. Data are presented as mean ± S.D. Statistical significance was determined using an unpaired *t*‐test, with a *p*‐value of < 0.05 considered significant.

We have demonstrated that monitoring gastric mucosal pH changes can precisely identify gastric cancer tissues. However, conventional algorithms require time‐consuming standard curve measurements. To achieve rapid and accurate pH mapping, we propose the application of advanced AI techniques (Figure S9, Supporting Information). We divided the 50 samples into two sets: one for AI training and validation (40 samples) and another for external validation (10 samples). Detailed patient data is presented in **Table** **1** and Table S1 (Supporting Information), while **Figure** **5**a depicts the operational principle and workflow of the AI model. The pH map acquisition time was notably reduced from more than 30 min to within 5 min (30 sampling points). Consistently, the pH values in tumor regions (7.105 ± 0.3573) are significantly higher than those in adjacent mucous membranes (6.078 ± 0.8273), with a critical pH threshold for differentiation established at 6.845 and an AUC of 95.13% (95% CI, 0.9386‒0.9639) (Figure 5b,c).

**Table 1:** Demographic characteristics.

Characteristics		
	Original cohort	External validation cohort
Number of specimens	40	10
**Number of points (T/N)**	1127(448/679)	389(127/262)
**Age, y, mean ± SD**	64.40 ± (9.84)	63.40 ± (11.43)
**Gender**		
Male, n (%)	29 (72.5%)	8 (80%)
Female, n (%)	11 (27.5%)	2 (20%)
**Location**		
cardia	5 (12.5%)	0 (0%)
Gastric body	13 (32.5%)	5 (50%)
Gastric antrum	22 (55%)	5 (50%)
**Degree of differentiation**		
Highly differentiated	29 (72.5%)	7 (70%)
High to moderately differentiated	10 (25%)	3 (30%)
Poorly differentiated	1 (2.5%)	0 (0%)

The accuracy of the AI model was validated by using an external validation set. We compared the deep learning model with the nonlinear regression model using R^2^ and SSE, which revealed an R^2^ of 0.79 and SSE of 71.83 for the external validation set (Figure 5d). Further analysis showed that the model exhibited sensitivity 85.04% (95% CI, 0.773‒0.9053), specificity 87.79% (95% CI, 0.8305‒0.9138), (positive predictive value) PPV 77.14% (95% CI, 0.6913‒0.8363) and (negative predictive value) NPV 92.37% (95% CI, 0.8816‒0.9522) in identifying tumor tissues vs para‐tumor tissues in 389 points of ESD samples, and its overall diagnostic accuracy reached 86.89% (95% CI, 0.8317‒0.8989) with a strong inter‐observer agreement (κ = 0.71) as shown in Table S2 (Supporting Information) and Figure 5e. These metrics demonstrate the effectiveness of our model in detecting pH values and its potential for accurate tumor predicting before pathological analysis, which generally takes 5‒7 days.

**Figure 6:**
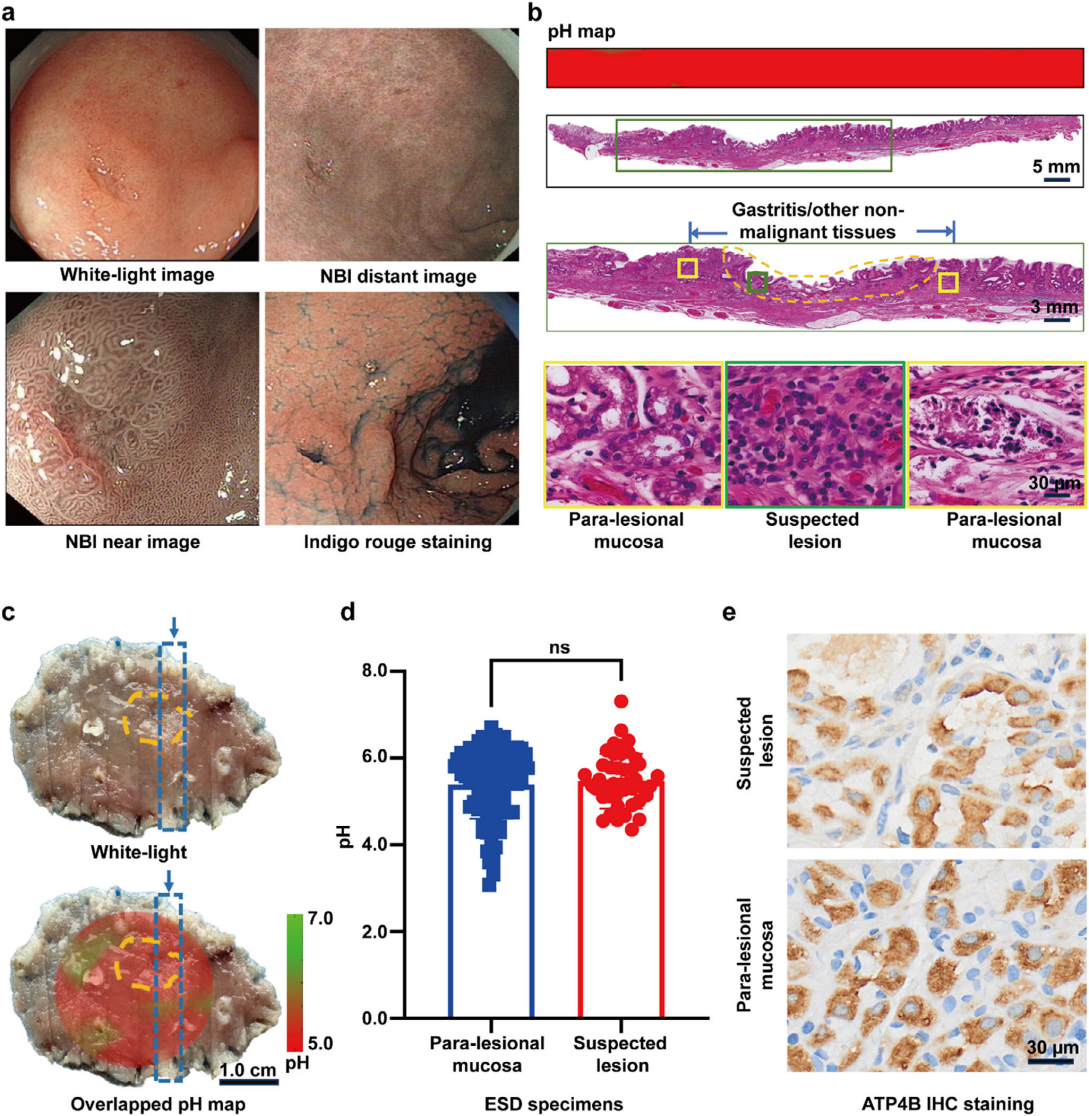
AI‐assisted SERS microarray identifies EGC from gastritis. a) In one of the cases, the differentiation between benign and malignant lesions has been challenging via endoscopic examination. Biopsy findings indicated neoplastic growth, but ESD confirmed benign pathology. b) pH map and H&E staining of the ESD strip highlighted in c (blue dotted box), showing uncompromised acidity in inflammatory tissue. The lower two panels show enlarged images. Green box: suspected lesion, yellow box: para‐lesional gastric mucosa. Scale bar: from top to bottom are 5 mm, 3 mm, and 30 µm. c) White light pathological restoration map (up) and the overlap of pathological restoration map and pH topographic map (down) for a case pathologically verified as gastritis. The yellow dotted lines highlight the suspected lesion. Scale bar: 1.0 cm. (d) pH values measured in suspected lesions and para‐lesional mucosae (*N* = 6, total points = 38, suspected vs para‐lesional = 45 vs 93, *p* = 0.5635). e) Immunohistochemical staining of ATP4B in the suspected lesion and para‐lesional mucosa. Scale bar: 30 µm. Data are presented as mean ± S.D. Statistical significance was determined using an unpaired *t*‐test, with a *p*‐value of < 0.05 considered significant.

To further verify the reliability of the AI model, we reviewed another 6 specimens that cannot be interpreted normally: the neoplastic specimens (total points = 138; suspected lesions vs para‐lesional mucosae: 45 vs 93) identified by endoscopy and biopsy pathology, but ultimately confirmed non‐neoplastic in ESD pathology (**Figure** **6**a,b). Detailed patient data is in Table S3 (Supporting Information). pH topographic maps were generated using an AI‐assisted SERS microarray system on fresh ESD specimens, showing no obvious acidity difference between suspicious lesions (marked with yellow dotted lines) and para‐lesional gastric mucosae (5.467 ± 0.6293 vs 5.389 ± 0.7858, *p* = 0.5635) (Figure 6c,d). In addition, there was no difference in ATP4B level seen in the non‐malignant lesions and para‐lesional tissues through immunohistochemical analysis (Figure 6e). This finding corroborates the model's reliability in identifying pH patterns and pathological features.

## 3 Discussion

Accurate diagnosis is fundamental to the detection and precise resection of EGC. Currently, the endoscopic morphological observation exhibits a significant misjudgment rate that cannot be ignored, and there are relatively large inter‐operator differences among individual doctors. Our study introduces a novel method for pinpointing EGC during ESD by mapping the pH levels of suspicious regions, including biopsy specimens. This method emphasizes the changes in gastric mucosal pathology and molecular biology, particularly using alterations in acidity as an indicator of malignancy. It collaborates with endoscopic morphological observation to facilitate early diagnosis and precise localization of gastric cancer both before and during ESD procedure, enabling precise resection and minimizing false positive rates.

The gastric ATP4 is composed of an α‐subunit (ATP4A, which contains the catalytic site for ATP hydrolysis)^[^ ^26^ ^]^ and a β‐subunit (ATP4B, which stabilizes the catalytic α‐subunit and mediates the final step of acid secretion).^[^ ^27^ ^]^ Intestinal gastric cancer carcinogenesis typically progresses from normal gastric mucosa through chronic non‐atrophic gastritis, chronic atrophic gastritis, intestinal metaplasia, and dysplasia before culminating in intestinal‐type EGC.^[^ ^28^ ^]^ During this progression, in alignment with our findings, the downregulation of *ATP4* mRNA, especially *ATP4B*, begins with pre‐cancerous lesions (especially intestinal metaplasia) and becomes undetectable in EGC.^[^ ^19^, ^29^ ^]^ Given the previous observation that the downregulation of *ATP4B* expression is closely related to low gastric acidity,^[^ ^20^ ^]^ this provides a theoretical foundation for precisely locating EGC through the detection of acidity compromise. Simultaneously, the pH threshold of 6.845 also enables us to differentiate malignant tissue from deeper layers exhibiting a neutral pH level, such as the outer muscularis and serosa.

Our multifaceted approach also addresses limitations noted in previous studies. First, while earlier research employed a fiber optic Raman system to detect EGC on excised ESD samples by monitoring amino acid Raman peaks,^[^ ^30^ ^]^ spontaneous Raman spectroscopy is limited by low signal intensity, long acquisition times, and sensitivity to tumor heterogeneity, compared to SERS.^[^ ^31^ ^]^ Furthermore, the ratio‐metric strategies improve the stability and accuracy of quantitative information by built‐in self‐calibration.^[^ ^32^, ^33^, ^34^ ^]^ Second, pH‐responsive nanoprobe advancements have shown effective intraoperative tumor localization through pH mapping.^[^ ^35^ ^]^ While these probes have accurately delineated tumor margins, their clinical approval may be delayed due to strict safety regulations. Our SERS microarray‐based strategy accelerates clinical translation potential by eliminating the need for exogenous probes. Third, our method offers clear advantages over traditional pH measurement techniques. For instance, electrode‐based pH measurements require tissue insertion. In contrast, our SERS microarray system utilizes a water droplet as a transfer medium, thereby minimizing invasiveness and tissue damage. Moreover, while microcapillary pH meters require sample volumes greater than 2.5 µL, our system accurately measures pH in droplets as small as 0.5 µL, enhancing spatial resolution. Conventional pH meters require 10‒15 s per measurement (excluding calibration), whereas our AI‐assisted system takes 1‒2 s, enabling rapid pH mapping during endoscopy. Finally, Raman spectroscopy can simultaneously visualize multiple tumor metabolites, including not only pH values but also various enzymatic activities,^[^ ^36^, ^37^, ^38^ ^]^ thereby enhancing the precision of malignant tissue localization.

Indeed, several early studies have integrated SERS with AI for the detection of gastric cancer/EGC, focusing on indirect specimens such as exhaled breath,^[^ ^39^, ^40^, ^41^ ^]^ serum,^[^ ^42^, ^43^ ^]^ ascites^[^ ^44^ ^]^ and small extracellular vesicle.^[^ ^45^ ^]^ While these biofluid‐based approaches can indicate tumor presence and even differentiate stages, they lack spatial localization within the stomach, making them unsuitable for real‐time endoscopic guidance. Additionally, studies like Liu et al. have used endoscopic biopsy tissues for diagnostic research, employing single‐shot femtosecond stimulated Raman scattering combined with an integrated U‐Net to identify structural features consistent with histopathology findings,^[^ ^46^ ^]^ and developing a convolutional neural network (CNN) for early gastric cancer prediction. However, their method relies heavily on morphological contrast, which often lags behind metabolic and molecular changes indicative of early malignancy.^[^ ^47^ ^]^ In contrast, our approach focuses on metabolic features, which may better delineate early gastric cancer boundaries where histological changes are minimal. Furthermore, while stimulated Raman scattering instrumentation is costly, technically demanding, and limited to ex vivo analysis, our portable and cost‐effective system simplifies operation and holds potential for in vivo, non‐invasive, or minimally invasive detection. Finally, our system achieves analysis within 1‒2 s, significantly faster than Liu et al.’s method (reported inference exceeding 1 min per site).

In this study, we developed a sophisticated multi‐model neural network to predict pH values. It integrates 1D Raman spectra sequences with 2D Raman spectral images, enhancing the model's ability to capture spectral details and spatial relationships. This enables a more comprehensive analysis that effectively leverages complementary information from different data sources to improve the accuracy and robustness of pH prediction. Additionally, a Co‐Attention mechanism further enhances this integration by dynamically focusing on the most relevant features in both modalities, assigning attention weights to sequences and images.

Despite these promising results, our study has several limitations. The relatively small cohort size necessitates further research with larger, more diverse samples to ensure long‐term accuracy and reliability. Additionally, the current system cannot differentiate between various pathological grades (high, moderate, or poorly differentiated) of EGC. While acidity mapping provides valuable insights, it captures only a fraction of the complex molecular landscape of gastric cancer. In the future, it may be possible to directly detect in vivo tissues, achieving non‐invasive or minimally invasive detection, and extending to scattered early‐stage lesions. Future research should integrate endoscopic morphological observations with comprehensive SERS fingerprinting and advanced AI‐driven visualization techniques. This integration holds promise for not only early diagnosis but also for pathological classification and assessment of infiltration depth‐paving the way for more efficient, precise, and AI‐enabled diagnostic and therapeutic strategies.

## 4 Conclusion

We have developed an AI‐assisted SERS microarray system that rapidly and accurately generates pH maps for biopsy and ESD specimens. Our analysis of ESD specimens revealed a critical pH threshold of 6.845 that distinguishes gastric tumors from para‐tumor mucosa. This innovative system holds the potential to transform the diagnosis and treatment of EGC by enabling more precise ESD procedures, reducing unnecessary excisions, enhancing resection rates, and supporting informed clinical decision‐making.

## 5 Experimental Section

### Study Design

The purpose of this study was to develop an AI‐assisted endoscopic bedside diagnostic system for precise localization of EGC using SERS microarray chips as a tool for rapid and accurate detection of gastric mucosal pH topography. To analyze the immunohistochemical markers of non‐EGC biopsy specimens, tissues were selected from individuals identified as non‐EGC cases through gastroscopic screening, as well as adjacent tissues from EGC specimens. Data obtained from public databases and clinical specimens were applied to analyze the expression levels of ATP4B. Based on power analysis, with α = 0.05, β = 0.2, and a 1:1 enrollment ratio (paired cancerous and adjacent tissues from the same ESD patient), it was determined that 10 ESD specimens diagnosed as EGC were required. Therefore, the feasibility of this approach was initially explored using 10 cases in an animal model. Our study primarily focused on high/moderately differentiated intestinal‐type gastric cancer, which comprised the majority of ESD‐treated EGCs in pathological classification. Patients who had contraindications for ESD or biopsy, or were unable to provide consent, were excluded from the study. Traditional algorithms were applied to evaluate the sensitivity, specificity, and accuracy of pH values in distinguishing between benign and malignant gastric mucosa. All animal and patient samples were compared to the pathological restoration maps. With the introduction of an AI model, samples were split between training, internal validation, and external validation, necessitating a minimum of 50 ESD specimens for EGC. Among them, 80% of samples were randomly allocated for training and validation purposes, with a specific distribution of 60% randomly selected for training and the remaining 20% for internal validation. The remaining 20% was reserved for external validation. Performance metrics, including R^2^, SSE, sensitivity, specificity, and accuracy, were calculated for the AI model, and the procedure was shown in Figure S9 (Supporting Information). Finally, conducted a further evaluation of the system's reliability using six inflammatory ESD lesions identified during the sample collection phase.

### Materials

HAuCl_4_·4H_2_O, sodium citrate·2H_2_O, aminopropyl triethoxy silane (APTES), NH_3_·H_2_O, 4‐(2‐hydroxyerhyl) piperazine‐1‐erhanesulfonic acid (HEPES), polyoxymethylene, and glass cover slips were purchased from General Reagent, Shanghai. The 4‐inch diameter silicon wafers were purchased from Shanghai Zhiyan Electronic Technology Co., Ltd. H_2_O_2_ (30%), Na_2_HPO_4_·12H_2_O (99.0%), citric acid·H_2_O (99.0%), and HCl (37.5%) were purchased from Sinopharm chemical reagent Co., Ltd. Ultrapure (Up) water was produced by a MT system (18.2 MΩ cm, Shanghai Leading Water Treatment Equipment Co., Ltd., China). RPMI1640, fetal bovine serum (FBS), D‐Luciferin sodium salt, and penicillin/streptomycin were purchased from Dalian Meilun Biology. All reagents were purchased from commercial sources and used without further purification.

### Patients and Tissue Specimens

In our primary study, a meticulous selection process took place at the Endoscopy Center of the Second Affiliated Hospital of Zhejiang University School of Medicine. Prior to participation, all patients provided written informed consent. Our study protocol was approved by the Ethics Committee of our hospital (No. 20230516) and strictly adhered to the ethical guidelines of the Declaration of Helsinki (1975).

### Cell Lines and Animal Models

The human gastric cancer cell lines NCI‐N87‐Luc (expressing luciferase), MKN‐45, and AGS were sourced from the American Type Culture Collection (ATCC) and cultured in RPMI 1640 medium with 10% FBS and penicillin/streptomycin at 37 °C, 5% CO_2_.

Male‐specific pathogen‐free (SPF) athymic nude mice, aged 6 weeks and weighing 18–20 g, were obtained from Shanghai Sippr‐BK Laboratory Animal Co. Ltd, and were maintained on a standard diet. All animal studies adhered to the ARRIVE guidelines. After a 24‐h fast, mice were anesthetized with avertin via intraperitoneal injection. Subsequently, the stomach was exposed, and 50 µL of cancer cells (1×10^6^ cells/mL) were injected into the submucosa. From day 5 post‐implantation, D‐Luciferin sodium salt was administered intraperitoneally for bioluminescence imaging to monitor tumor growth every two days. Model mice that were not successfully modeled or whose tumors reached or broke through the muscularis mucosa were excluded, as EGC was located in the mucosal and submucosal layers.

### Fabrication of SERS Microarray

The synthesis of SERS microarray follows a method by Ziyi Jin,^[^ ^32^ ^]^ involving several steps. First, silicon wafers were cleaned with a solution of H_2_O, NH_3_·H_2_O, and H_2_O_2_ (5:1:1) at 80 °C for 30 min to reveal surface hydroxyl groups. Then they were functionalized with 2% APTES in ethanol for 12 h. Gold nanospheres (45 nm) were created using a 24 × 10^−3^ HAuCl_4_ solution and 1% trisodium citrate dihydrate. These nanospheres coat the functionalized wafers and were incubated in a solution of 70 × 10^−3^ M HEPES and 0.5 × 10^−3^ M HAuCl_4_ at 10 °C to form short branches. The wafers were then immersed in an IR7p methanol solution for 12 h. For analysis, buffer solutions with different pH values were applied, and their spectra were collected using a portable spectrometer equipped with a 785 nm laser, a 600‐gr mm^−1^ grating, and an acquisition time of 500 ms. The intensity ratios of specific peaks at 303 cm^−1^ and 520 cm^−1^ demonstrate a linear relationship with pH.

### Animal Studies

In an SPF‐level lab, a mouse model was anesthetized via intraperitoneal injection of 1.25% avertin for surgery involving incision, exposure of abdominal cavity and stomach, and tumor excision with surrounding normal tissues. Immediately after excision, the tissue surface was gently rinsed with saline to remove gastric acid (low‐pH gastric mucus) or other contaminants, wiped the specimen surface with gauze to remove the saline, and detected the pH value after 1 min. A pipette was used to apply 0.3 µL of ultrapure water onto the excised tissue surface for 2.5 s, creating a water droplet with dissolved metabolites. This droplet was then transferred to a SERS microarray for Raman analysis. Sampling points were spaced at 3 mm intervals, and pH values were calculated to generate a pH map. Our study protocol was approved by the Ethics Committee of our hospital (No. 2022034).

### Location of Human EGC on ESD Specimens

All procedures of endoscopic screening and ESD were performed with high‐definition scopes (EVIS LUCERA ELITE CV‐290 processor, 290 series gastroscope; Olympus, Tokyo, Japan). A 3mm×3 mm sampling grid was used to standardize points on human gastric ESD specimens. Measurement procedures mirrored animal studies, with rapid data acquisition in 5 min. Samples were then fixed in paraformaldehyde for H&E and (Immunohistochemistry) IHC staining. To measure pH standard curve and analyze the Raman spectra, an additional 30 min or more was required. 50 patient specimens were collected, and the details were shown in Table S1 (Supporting Information). The critical pH value was determined using a ROC curve to distinguish tumor from para‐tumor tissue.

### AI‐Assisted pH Topographic Mapping, the Training and Validation Set

In this study, a multi‐model network was designed to predict pH values by combining features from 1D Raman spectral sequence and 2D Raman spectral images. Specifically, Raman spectra were converted into 3 types of 2D images: RP, GASF, and GADF. Features were extracted using pre‐trained ResNet‐18. It applies convolutional operations to get multi‐level features. Residual connections help solve the vanishing gradient, making it suitable for complex feature extraction. To adapt to our task, the final fully connected layer of ResNet‐18 was removed, allowing the extracted features to serve as inputs for the subsequent module. For 1D spectral data, a fully connected layer was used. The Co‐Attention mechanism efficiently models interactions across various modalities by focusing on key elements of each input. In our application, the Co‐Attention layer utilizes 1D spectral data features as queries, directing the integration of 2D spectral image features into a comprehensive representation through calculated co‐attention weights. The details of AI model establishment were available in the Supporting Information.

The Raman spectrum dataset was meticulously divided into an 80% training and validation set (*N* = 40, total points = 1127), and a 20% external validation set (*N* = 10, total points = 389) for AI‐driven analysis. This approach obviates the need for collecting standard curves. With the acquisition of all Raman spectra from the specimen, generating a pH topographic map of the ESD specimen now takes just 1–2 min. The critical pH value for the AI‐assisted system was also determined using an ROC curve to distinguish tumor from para‐tumor tissue.

### External Validation Set

The malignancy of 10 randomly selected ESD samples was independently evaluated using a conventional algorithm and an AI model, generating pH maps for tumor locations, and the performance of the AI model compared to the conventional algorithm was assessed using the R^2^ and the SSE. Post‐resection pathological examination was conducted, and the specimens were reconstructed. By comparing AI‐generated pH maps and pathological tumor inversion maps in 10 lesions, various diagnostic parameters such as sensitivity, specificity, positive PPV, NPV, and accuracy were also calculated.

### Histopathological Staining

H&E staining was performed on paraffin‐embedded sections from human and mouse specimens. Immunohistochemical staining was conducted using a primary antibody against ATP4B (SANTA CRUZ, sc‐374094), diluted at a ratio of 1:200. The sections were subsequently incubated with a secondary goat anti‐rabbit antibody (Servicebio, G1301‐10 mL) at room temperature. After staining, washing, and dehydration procedures, the sections were scanned using an Olympus BX51 microscope and observed by at least two pathologists who were unaware of the pH results for confirmation.

### Statistical Analysis

Origin 2022 software (Microcal Software Inc., Northampton, USA) and GraphPad Prism 8.0 were utilized for statistical analysis. Data were expressed as the mean ± standard deviation of all results. Statistical differences between the two groups were analyzed using a two‐tailed Student's *t*‐test. ANOVA was employed for multiple comparisons. A *p*‐value of <0.05 was considered statistically significant. Before data statistics, use the Shapiro‐Wilk test to assess the normality of the data and the Levene test to check the homogeneity of variance.

## Conflict of Interest

The authors declare no conflict of interest.

## Supporting information



Supporting Information

Supporting Data

## Data Availability

The data that support the findings of this study are available from the corresponding author upon reasonable request.
