# Supplementary material for: AI‐Assisted Detection of Early Gastric Cancer via Visualization of Mucosal Acidity Compromise During Endoscopy
Source: Adv Sci (Weinh). 2025 Oct 20;12(46):e04932. doi: 10.1002/advs.202504932 (PMC12697849; doi:10.1002/advs.202504932)
Supplement: Supplementary file 1 — Supporting Information [file ADVS-12-e04932-s001.docx]

Supporting Information

AI-Assisted Detection of Early Gastric Cancer via Visualization of Mucosal Acidity Compromise During Endoscopy

Authors：Huihui Yan^1†^, Zongkuo Li^1†^, Jing Zhao^2†^, Lei Su^3†^, Ziyi Jin^2†^, Weiyi Zhao^4^, Rong Duan^4^, Suhongrui Zhou ^2^, Lingling Wang^1^, Jianshan Mao^1^, Xinliang Lu^1^, Weihao Gai^3^, Yang Du^3^, Qin Du^1^, Cheng Fang^1^, Yiming Zhao^5^, Yan You ^2,6^*, Jianting Cai^1^*, Cong Li^2^*

Affiliations：

^1^Department of Gastroenterology, The Second Affiliated Hospital, Zhejiang University School of Medicine; Hangzhou, China. E-mail: [jtcai6757@zju.edu.cn](mailto:jtcai6757@zju.edu.cn)

^2^MOE Key Laboratory of Smart Drug Delivery, MOE Innovative Center for New Drug Development of Immune Inflammatory Diseases, State Key Laboratory of Medical Neurobiology, School of Pharmacy, Fudan University; Shanghai, China. E-mail: [congli@fudan.edu.cn](mailto:congli@fudan.edu.cn); yyou@fudan.edu.cn.

^3^CAS Key Laboratory of Molecular Imaging, Institute of Automation, Chinese Academy of Sciences; Beijing, China

^4^Department of Pathology, The Second Affiliated Hospital, Zhejiang University School of Medicine; Hangzhou, China.

^5^Department of Child Healthcare, Jiangbei District Maternal and Child Health and Family Planning Service Center; Ningbo, China.

^6^Department of Pharmacology, School of Pharmacy, Fudan University; Shanghai, China. E-mail: yyou@fudan.edu.cn.


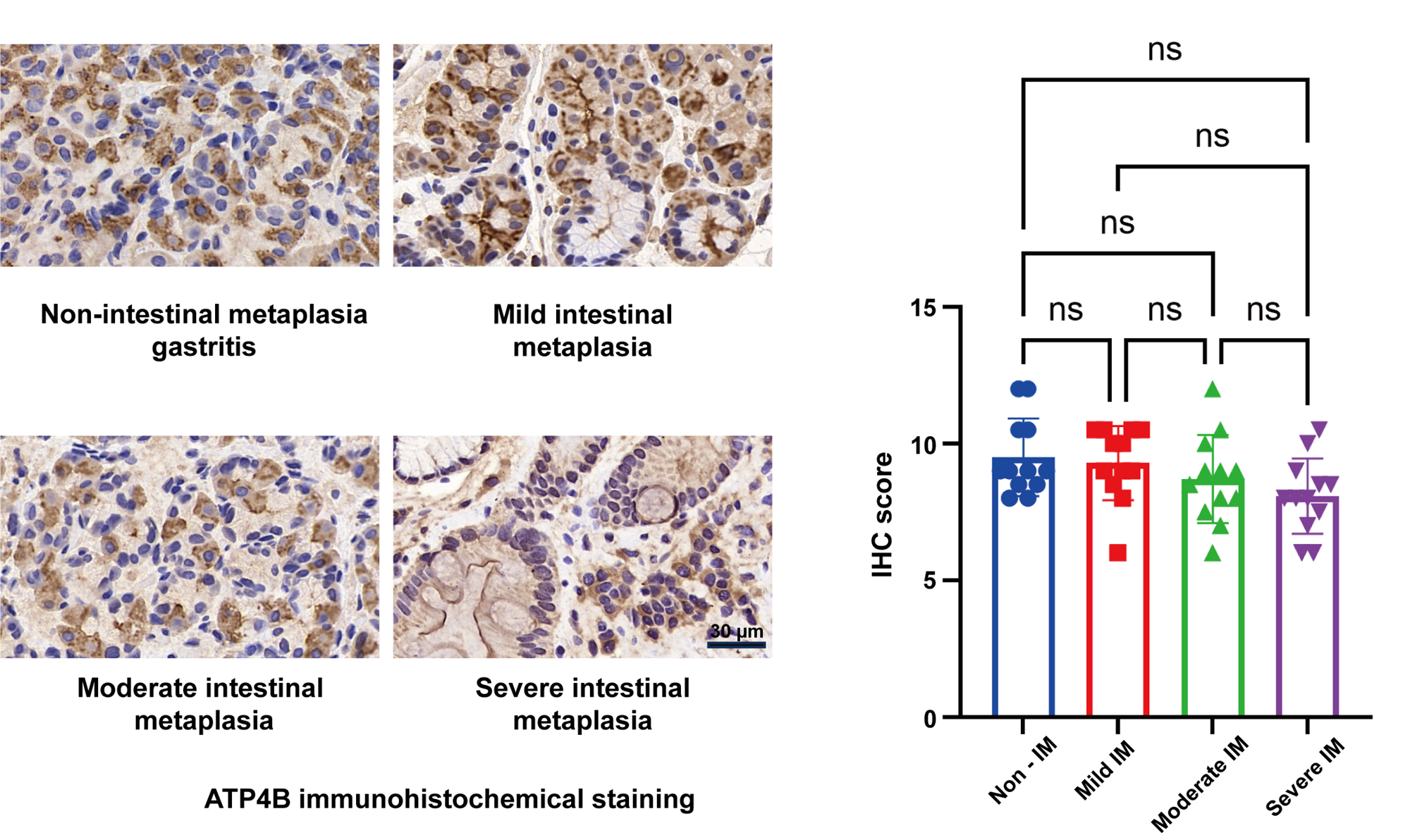


Figure S1. Comparison of ATP4B (ATPase H+/K+ transport β subunit) levels in patients' gastric mucosal biopsy specimens.

Scal bar: 30μm. Data are presented as mean ± S.D. Statistical significance was determined using one-way analysis of variance (ANOVA), with a *p*-value of < 0.05 considered significant.


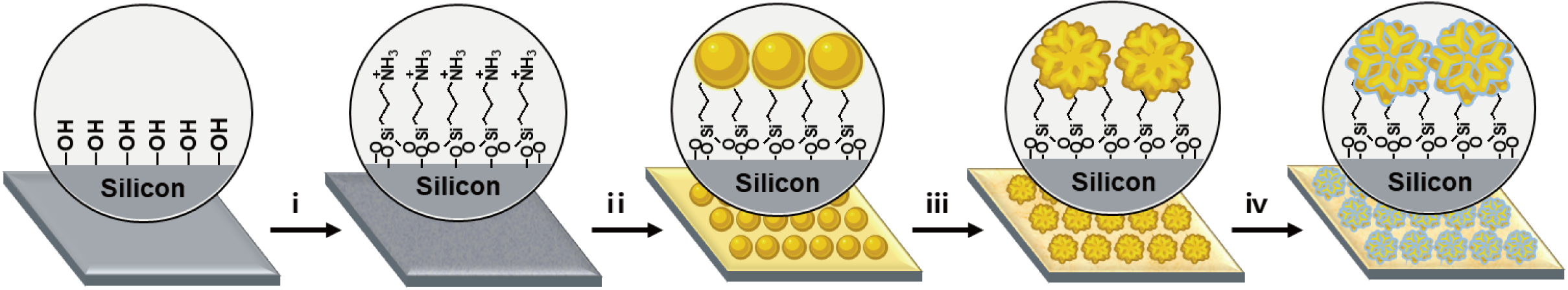


Figure S2. The preparation of pH ratio-metric SERS (surface-enhanced Raman scattering) microarray.

It begins with the functionalization of the silicon wafer surface with primary amines, followed by conjugation with gold nanorods with an average diameter of 45 nm. Subsequently, the gold nanospheres grow into nano-stars with an average diameter of 70 nm. Finally, the nano-stars are functionalized with the pH responsive Raman reporter IR7p. ⅰ) 2% (v/v) 3-aminopropyl-triethoxysilane in ethanol, 0.2 M HCl; ⅱ) gold nanospheres; ⅲ) 3 × 10^−4^ M HAuCl_4_ in 7.5 ×10^−2^ M HEPES solution (pH 7.4); ⅳ) 5 × 10^−6^ M IR7p solution. Raman reporter IR7p modified on the gold nano-star surface leads to the pH responsive Raman spectra.


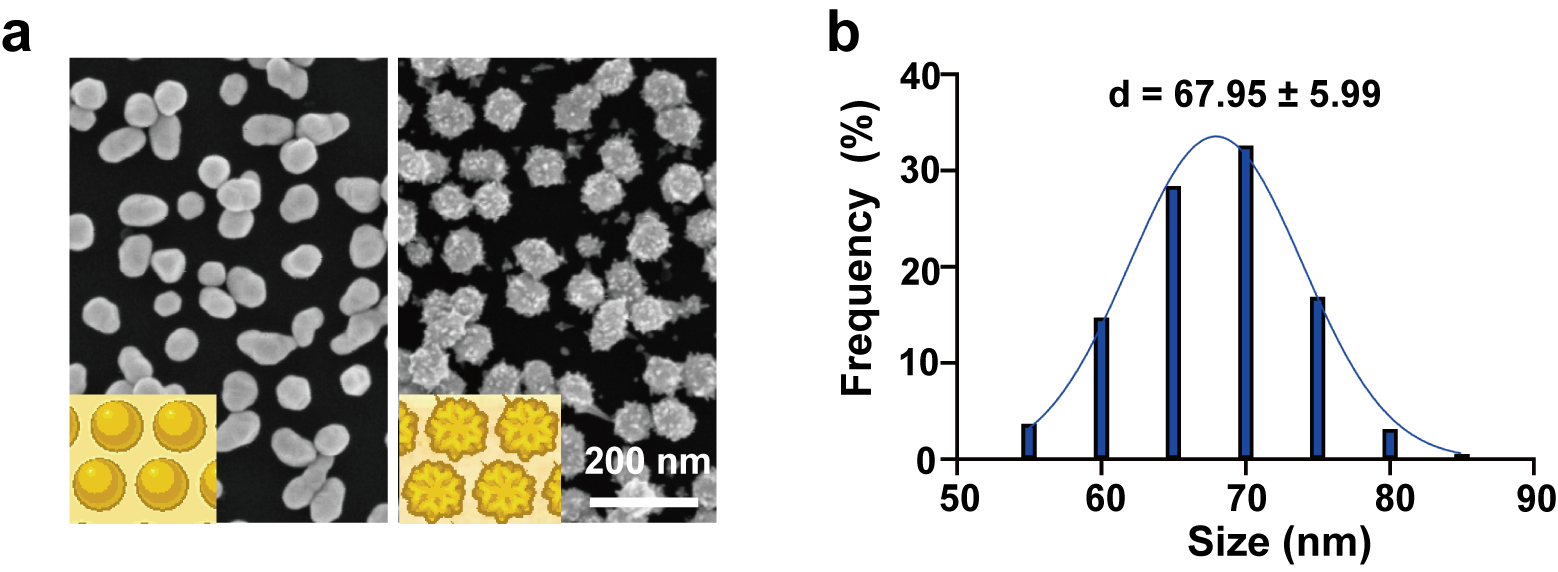


Figure S3. Scanning electron microscopy (SEM) images.

(a) The fabricated gold nanospheres and nano-stars on silicon wafer surfaces, with good uniformity. Scale bar: 200 nm. (b) The diameter of the gold nanostars is 67.95 ± 5.99 nm, corresponding to a relative standard deviation (RSD) of less than 9%, and the spatial distribution is 101.2 ± 5.391/μm^2^.


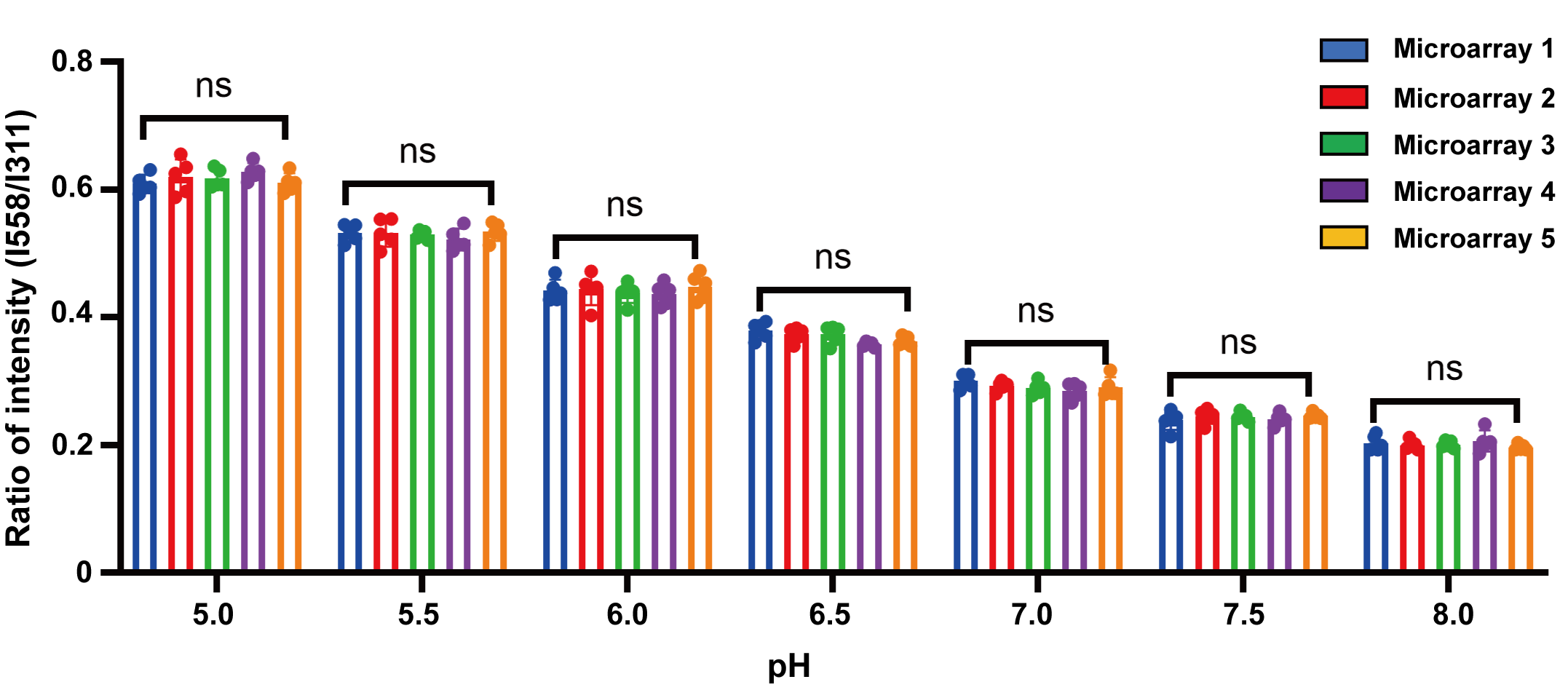


Figure S4. Evaluation of the batch-to-batch repeatability of SERS microarray chips in response to solutions of different pH levels.

Each color represents a chip from one of the five separative batches. No significant differences were observed in the detection values for the same pH standard solution across different batches. Data are presented as mean ± S.D. Statistical significance was determined using ANOVA, with a *p*-value of < 0.05 considered significant.


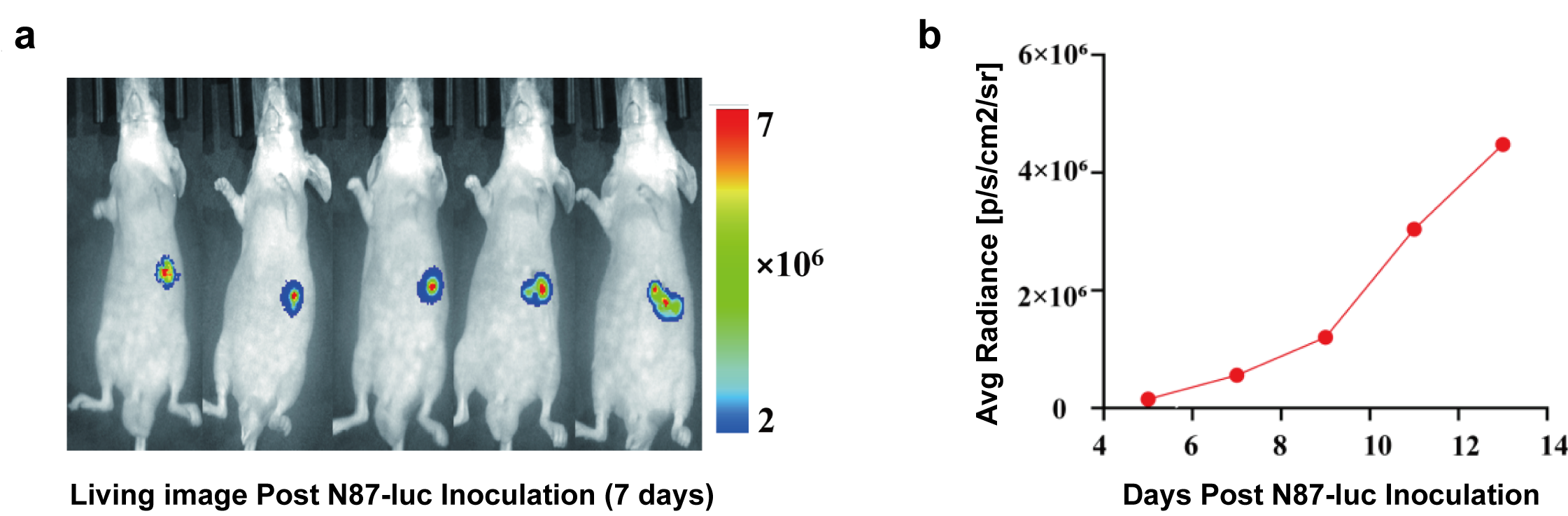


Figure S5. Establishment of in situ xenograft models of gastric cancer in nude mice.

(a) Fluorescence imaging after intraperitoneal injection of sodium fluorescein. (b) Tumor growth curve in vivo after NCI-N87-luc injection.


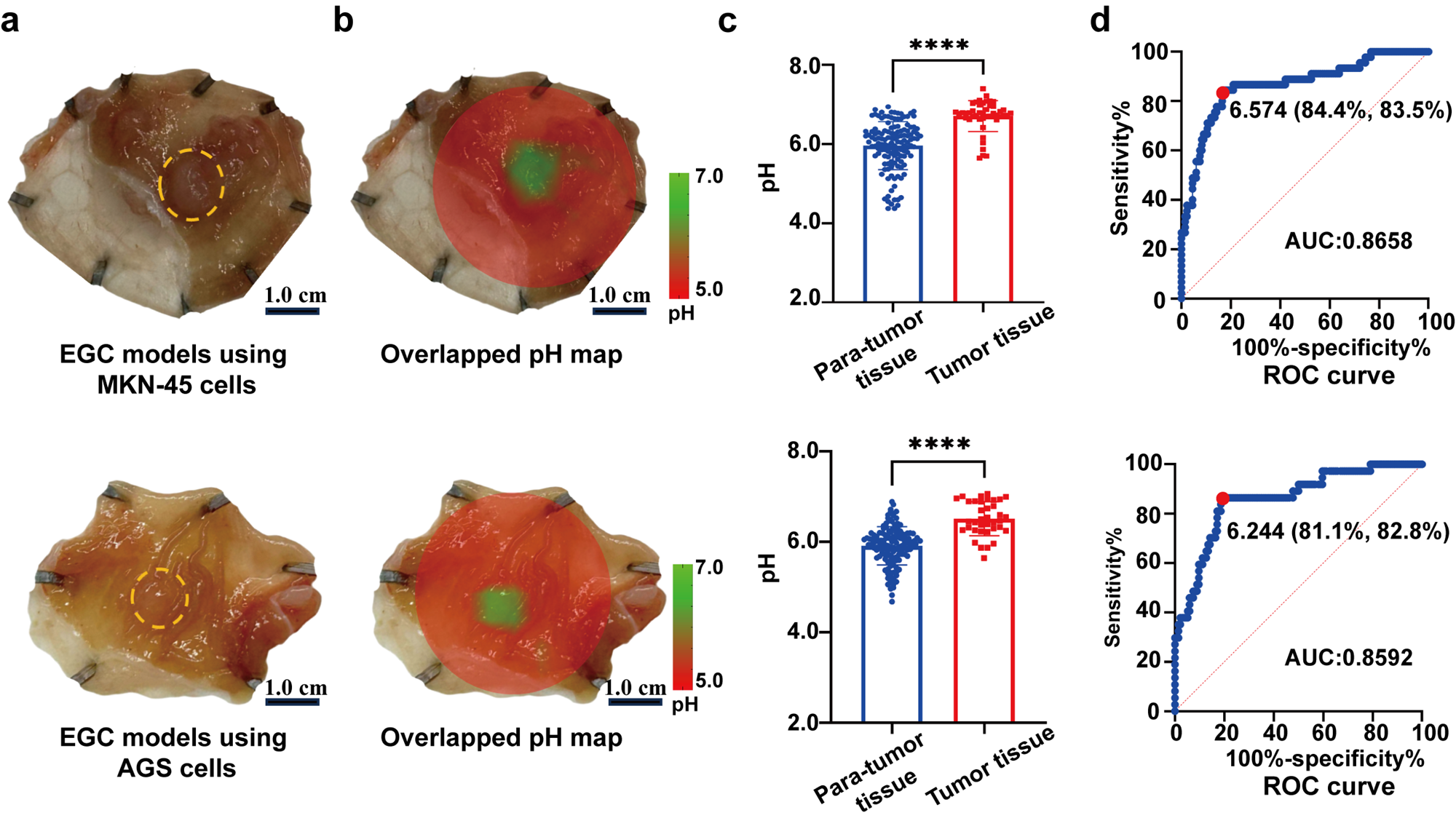


Figure S6. SERS microarrays accurately identify in-situ EGC (early gastric cancer) xenografts in tumor mouse models established using different gastric cancer cell lines.

Representative SERS mapping and diagnostic results demonstrate the robustness and reliability of the system across tumor models constructed with MKN-45 and AGS cell lines. Top row: MKN-45 gastric cancer cell line xenografts; bottom row: AGS gastric cancer cell line xenografts. (a) White light image showing EGC (indicated by a yellow dotted circle). Scale bar: 1.0 cm. (b) Overlaid pH maps of the excised EGC specimens. Scale bar: 1.0 cm. (c) pH measurements of the tumor and surrounding gastric tissue in excised specimens (MKN-45: n = 6, total points = 178; tumor vs. para-cancer: 45 vs. 133, *p* < 0.0001; AGS: n = 6, total points = 171; tumor vs. para-cancer: 37 vs*.* 134, *p* < 0.0001). (d) ROC (receiver operating characteristic) curve analysis for determining the optimal pH threshold to distinguish tumor tissue. The optimal threshold for MKN-45 xenografts is 6.574, with a sensitivity of 84.4% (95%CI, 0.7122‒0.9225), specificity of 83.5% (95%CI, 0.7622‒0.8882), and an AUC of 86.58% (95% CI: 0.7989‒0.9327). The optimal threshold for AGS xenografts is 6.244, with a sensitivity of 81.1% (95%CI, 0.6580‒0.9052), specificity of 82.8% (95%CI, 0.7556‒0.8828), and an AUC of 85.92% (95% CI: 0.7898‒0.9286). Data are presented as mean ± S.D. Statistical significance was determined using an unpaired *t*-test, with a *p*-value of < 0.05 considered significant.


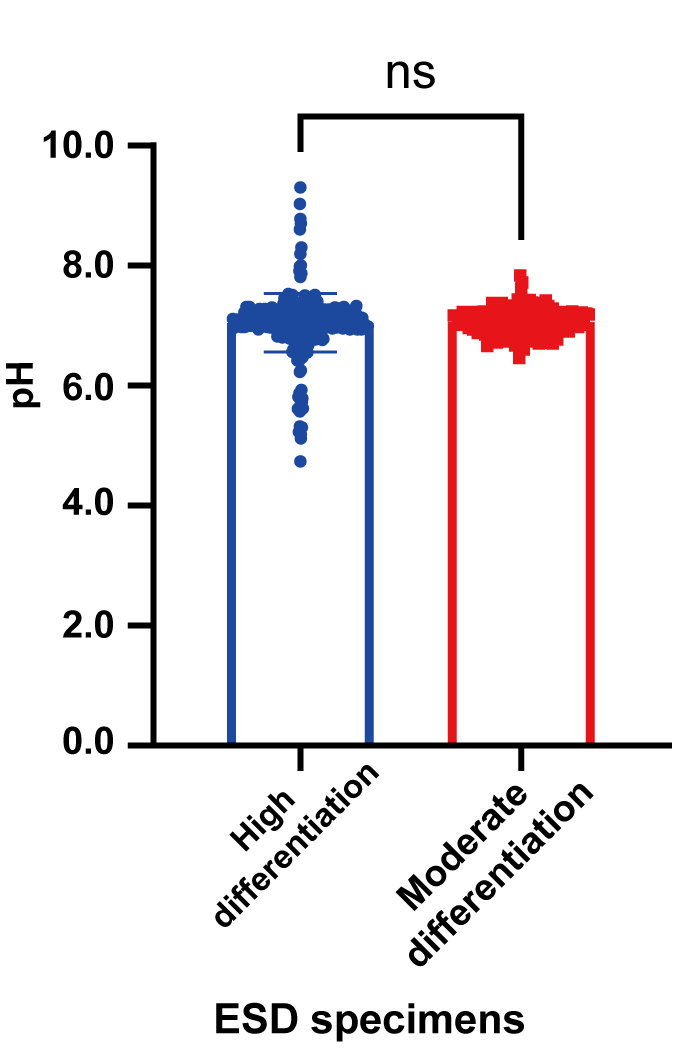


Figure S7. The pH values of well-differentiated and moderately differentiated EGC specimens.

There was no significant difference in tumor region pH between highly differentiated and moderately differentiated EGC specimens. Data are presented as mean ± S.D. Statistical significance was determined using an unpaired *t*-test, with a *p*-value of < 0.05 considered significant.


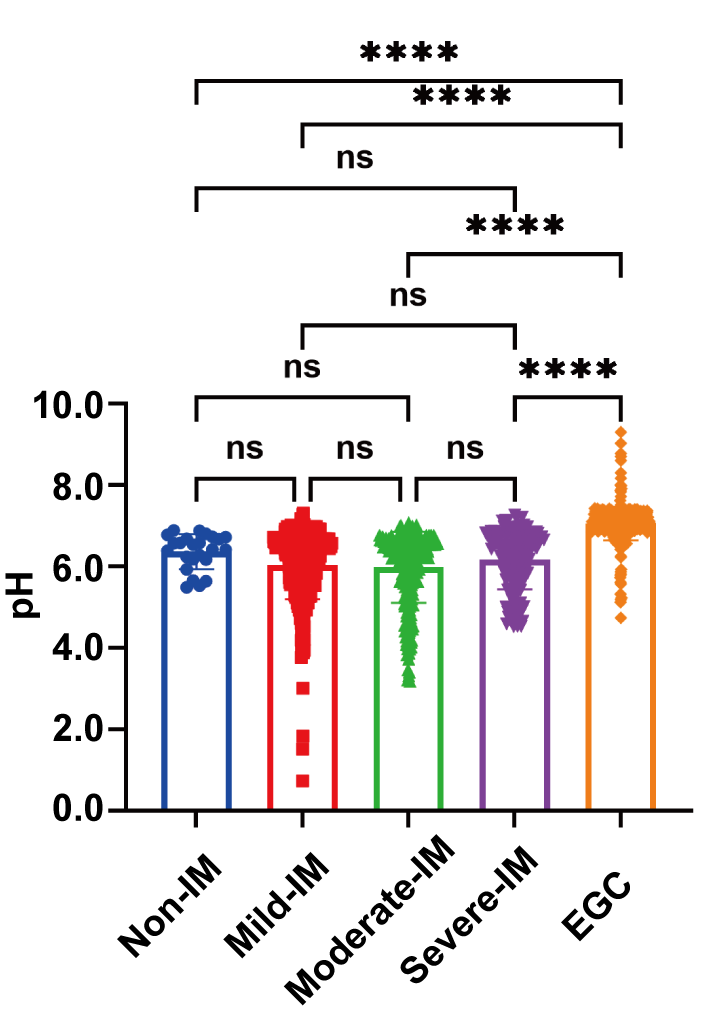


Figure S8. Comparison of pH values between background gastric mucosa and EGC sites in patients.

No significant differences in pH values were detected among the different degrees of atrophic gastritis with intestinal metaplasia. However, a significant increase in pH values was observed at EGC sites. Data are presented as mean ± S.D. Statistical significance was determined using ANOVA, with a *p*-value of < 0.05 considered significant.


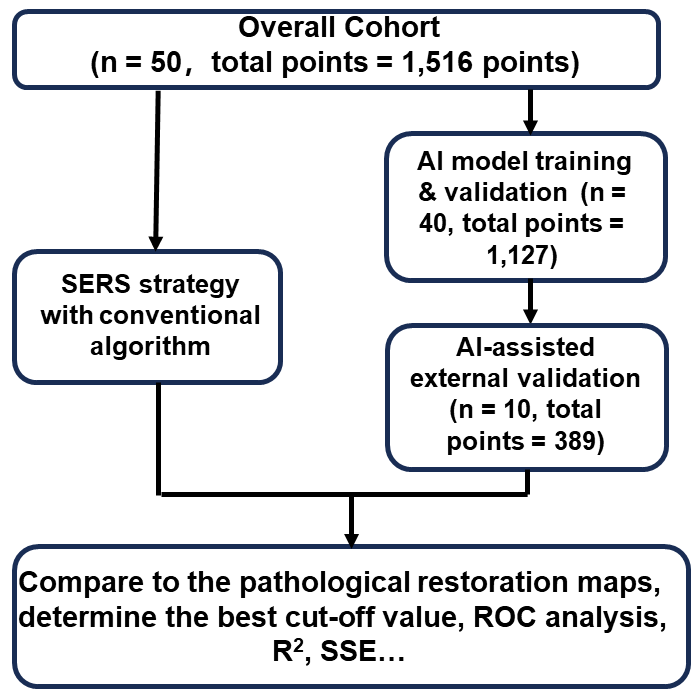


Figure S9. ESD specimen analysis procedure with AI (artificial intelligence) for EGC patients.

Table S1. Demographics characteristics of the EGC patients

|  | Early gastric cancer (n=50) |
| --- | --- |
| Number of points (T/N) | 1516(575/941) |
| Average age (SD) | 64.20 ± (10.05) |
| Gender |  |
| Male | 37 (74%) |
| Famale | 13 (26%) |
| Location |  |
| cardia | 5 (10%) |
| Gastric body | 18 (36%) |
| Gastric antrum | 27 (54%) |
| Grade of differentiation |  |
| Highly differentiated | 36 (72%) |
| High to medium differentiation | 13 (26%) |
| Poorly differentiation | 1 (2%) |

Table S2. Diagnostic ability of AI model

|  | Final Diag+ | Final Diag- |  |  |
| --- | --- | --- | --- | --- |
| AI Diag+ | 108 | 32 | PPV | 77.1% |
| AI Diag- | 19 | 230 | NPV | 92.3% |
|  | Sensitivity | Specificity |  |  |
|  | 85.0% | 87.8% |  |  |
| Accuracy=86.9% | | | | |
| κ=0.71 | | | | |

PPV: specificity, positive predictive value; NPV: negative predictive value

Table S3. Demographics characteristics of the gastritis patients.

|  | Gastritis patients. (n=6) |
| --- | --- |
| Average age (SD) | 54.17 ± (10.30) |
| Gender |  |
| Male | 2 (33%) |
| Famale | 4 (67%) |
| Location |  |
| cardia | 0 (0%) |
| Gastric body | 2 (33%) |
| Gastric antrum | 4 (67%) |

Artificial Intelligence Appendix

A total of 1,516 Raman spectra, each spanning a Raman shift range of 224 starting from the 15th coordinate, were used to predict pH values. For preprocessing, the raw spectral data were transformed into three 2D representations-Recurrence Plot (RP), Gramian Angular Summation Field (GASF), and Gramian Angular Difference Field (GADF)-which were then combined into three-channel images.

For feature extraction, a fully connected layer processed the 1D spectral data, while a pre-trained ResNet-18 backbone extracted hierarchical features from the 2D images. To fuse multimodal features, a Co-Attention mechanism was introduced. This mechanism, implemented as a single-layer, single-head attention module with an embedding dimension of 256, utilized 1D spectral features as queries to compute attention weights for the 2D image features, dynamically highlighting informative regions across modalities. The weighted features were then aggregated into a unified representation for pH prediction.

The deep learning model was trained end-to-end using PyTorch on an NVIDIA GeForce RTX 4090 GPU. The loss function was defined as mean squared error, and optimization was performed using the AdamW algorithm with an initial learning rate of 1e-4 and L2 regularization. Training was conducted for 1,000 epochs with a batch size of 8, using 4 parallel workers for data loading.
